# Prevalence of metabolic syndrome and associated factors among patient with type 2 diabetes mellitus in Ethiopia, 2023: asystematic review and meta analysis

**DOI:** 10.1186/s12889-024-18580-0

**Published:** 2024-04-23

**Authors:** Betelhem Mesfin Demissie, Fentaw Girmaw, Nimona Amena, Getachew Ashagrie

**Affiliations:** 1 Department of Nursing, School of Nursing, College of Health Sciences, Woldia University, North Wollo, Amhara, Ethiopia; 2 Department of Pharmacy, College of Health Sciences, Woldia University, North Wollo, Amhara, Ethiopia; 3 Department of Nursing, College of Health Sciences, Ambo University, Oromia, Ethiopia

**Keywords:** Metabolic syndrome, Type II diabetes mellitus, Prevalence, Ethiopia

## Abstract

**Background:**

Metabolic syndrome is a complex pathophysiologic state which characterized by abdominal obesity, insulin resistance, hypertension, and hyperlipidaemia. The Adult Treatment Panel III report (ATP III) of the National Cholesterol Education Programme identified the metabolic syndrome as a serious public health issue in the modern era. In Western and Asian nations, the frequency of metabolic syndrome is rising, especially in developing regions experiencing rapid socio-environmental changes, in Sub-Saharan Africa; metabolic syndrome may be present in more than 70% of people with type 2 diabetes mellitus. Therefore the objective of our study was to estimate the pooled prevalence of metabolic syndrome and associated factors among type II diabetes mellitus patient.

**Method:**

This systematic review and meta-analysis included original articles of cross sectional studies published in the English language. Searches were carried out in PubMed, Web of Science, Google Scholar, and grey literature Journals from 2013 to June 2023. A random-effects model was used to estimate the pooled prevalence of metabolic syndrome among type II Diabetes mellitus patient in Ethiopia. Heterogeneity was assessed using the I^2^ statistic. Subgroup analysis was also conducted based on study area. Egger’s test was used to assess publication bias. Sensitivity analysis was also conducted.

**Results:**

Out of 300 potential articles, 8 cross sectional studies were included in this systematic review and meta-analysis study. The pooled prevalence of metabolic syndrome among patient with type II diabetes mellitus in Ethiopia was found to be 64.49% (95% CI: 62.39, 66.59) and 52.38% (95% CI: 50.05, 54.73) by using NCEP/ATP III and IDF criteria, respectively. The weighted pooled prevalence of metabolic syndrome among type II diabetes mellitus patients by sub group analysis based on the study region was 63.79% (95% CI: 56.48, 71.11) and 52.23% (95%CI: 47.37, 57.22) by using NCEP/ATP III and IDF criteria, respectively. Being female and increased body mass index were factors associated with metabolic syndrome among type II diabetes mellitus patients.

**Conclusion:**

The prevalence of metabolic syndrome among type II patient is high. Therefore, policymakers, clinicians, and concerned stakeholders shall urge effective strategies in the control, prevention, and management of metabolic syndrome among type II diabetes mellitus.

## Introduction

The complicated pathophysiologic condition known as the metabolic syndrome is characterised by insulin resistance, hypertension, hyperlipidaemia, and abdominal obesity and which originate primarily from an imbalance between energy expenditure and calorie intake [1]. Even though the NCEP-ATPIII, IDF, and WHO criteria are the most often utilised clinical criteria for the diagnosis of metabolic syndrome, there are numerous similarities between them, there are also notable differences in the perspectives on the underlying causes of the metabolic syndrome [2]. The prevalence of metabolic syndrome is increasing in Western and Asian countries, particularly in emerging areas that are undergoing fast socio-environmental change. Numerous studies have demonstrated that metabolic syndrome is a major risk factor for type 2 diabetes mellitus, cardiovascular disease (CVD), and overall mortality [3].

Global estimates suggest that about one-third of the world’s population, primarily in developing countries, may have metabolic syndrome [4]. The National Cholesterol Education Programme’s Adult Treatment Panel III report (ATP III) recognised metabolic syndrome as a significant contemporary public health concern. It is a multiplex risk factor for cardiovascular disease (CVD) that requires more therapeutic care, and it also raises the risk of cancer, mental problems, renal illness, and early mortality [2, 5].

Compared to those in Western countries, the urban population in several developing nations has a greater prevalence of metabolic syndrome. The two main causes of this disease’s growth are the increase in fast food consumption—high-calorie, low-fiber foods—and the decline in physical activity brought on by sedentary leisure activities and the use of automated transportation [1].

In Sub-Saharan Africa, metabolic syndrome may be present in more than 70% of people with type 2 diabetes mellitus. According to research which is done in two rural clinics of Ghana, the prevalence of metabolic syndrome among type II diabetes mellitus patients was 68.6% (95% CI: 64.0-72.8), and having diabetes for more than five years, being female, and being overweight are significantly associated with metabolic syndrome [4]. A study done by Lira Neto JCG et al., Stated that among 201 study participants, 50.7% were diagnosed with metabolic syndrome [5].

A cross sectional study conducted in Ethiopia reported that the prevalence of metabolic syndrome was 20.3% among 325 study participants [6]. A Systematic Review and Meta-analysis study conducted in Ethiopia stated that the pooled prevalence of metabolic syndrome in Ethiopia was found to be 34.89% (95% CI: 26.77, 43.01) and 27.92% (95% CI: 21.32, 34.51) by using NCEP/ATP III and IDF criteria, respectively. Subgroup analysis based on the study subjects using NCEP/ATP III showed that the weighted pooled prevalence was 63.78%(95% CI: 56.17, 71.40) among type 2 Diabetes Mellitus patients [7].

Even though this topic has been studied before, our work is unique since we look at aspects outside prevalence. Thus, our study’s goal was to analyse the pooled prevalence of metabolic syndrome and associated factors among patients with type II diabetes mellitus. How much is the prevalence of metabolic syndrome among Ethiopian patients with type II diabetes mellitus? And what variables are associated with metabolic syndrome patients with type II diabetes in Ethiopian?

## Methods

### Protocol and search strategy

**Fig. 1:**
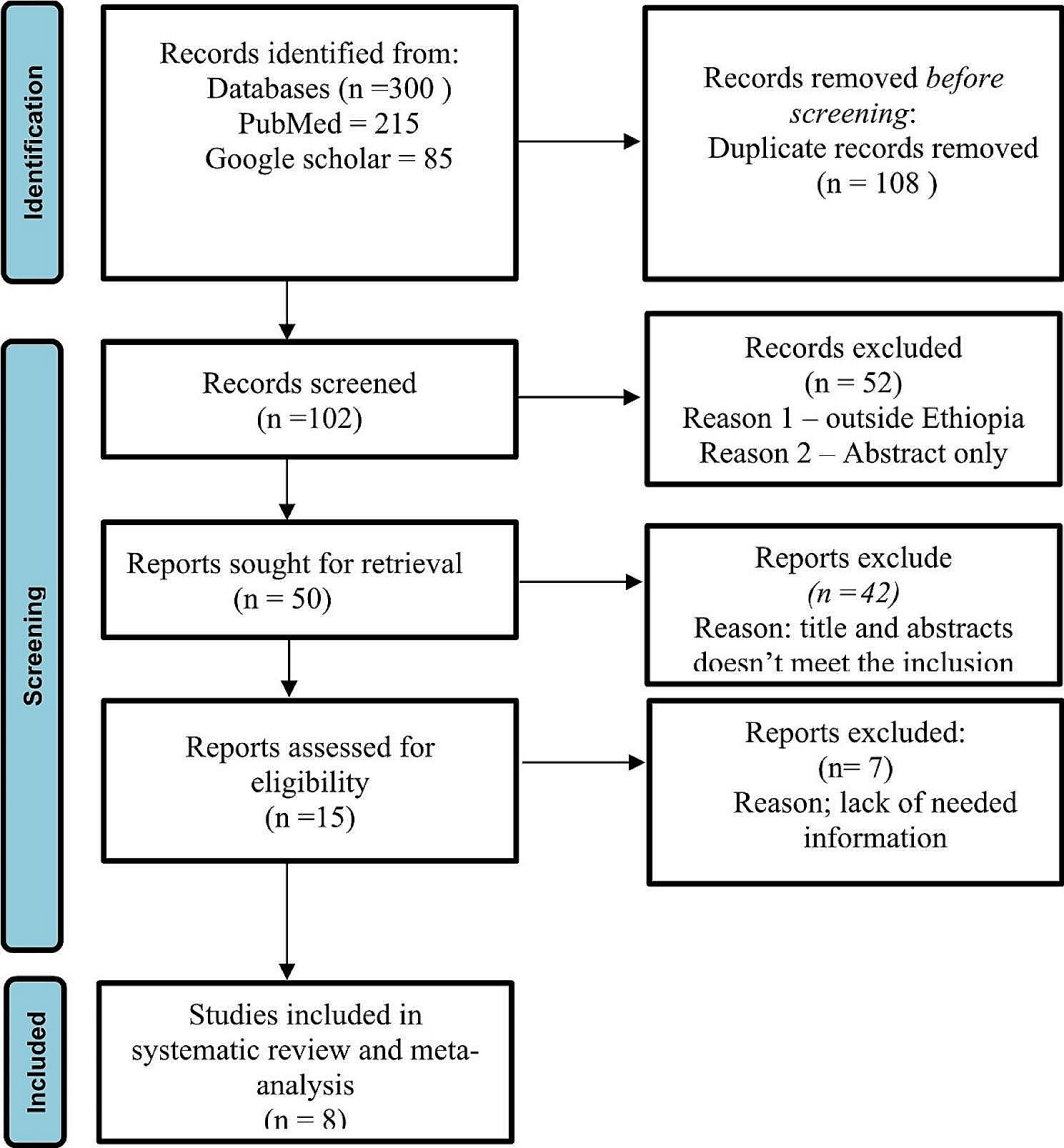
PRISMA flow diagram of study selection for systematic review and meta-analysis of prevalence of metabolic syndrome among type II Diabetes mellitus patients in Ethiopia [11]

The systematic review and meta-analysis was reported according to the Preferred Reporting Items for Systematic Reviews and Meta-Analyses (PRISMA) statement guideline (Fig. 1). The study protocol was registered in the PROSPERO International Prospective Register of Systematic Reviews (CRD42023442704). An inclusive literature search was conducted to identify studies about the prevalence of metabolic syndrome among patients with type II diabetes mellitus reported among the Ethiopian population of various study subjects. Both electronic and gray literature searches were carried out systematically. PubMed, Web of Science, Google Scholar, and grey literature, were searched for material between 2013 and 2023. The search terms were used separately and in combination using Boolean operators like “OR” or “AND.” An example of keywords used in PubMed to select relevant studies was as follows: (((“Metabolic Syndrome“[Mesh] OR Metabolic syndrome*[tiab]) AND (“Diabetes Mellitus, Type 2“[Mesh] OR “Diabetes Mellitus, Type 2*“[tiab])) AND (“Prevalence“[Mesh] OR “Magnitude*“[tiab])) AND (“Ethiopia“[Mesh] OR “Ethiopia*“[tiab]). Moreover, each database’s specific search parameters were customized accordingly.

### Study selection (inclusion and exclusion criteria)

#### Inclusion criteria

Studies were selected according to the following criteria: study design, participants, exposures and condition or outcome(s) of interest. Eligible studies were only quantitative full- text, and observational studies (cross-sectional) reporting prevalence and associated factors in terms of the odds ratio. Only articles written in English were retrieved for review. We included studies involving only type II diabetes mellitus patients aged greater than 30.

The primary outcome was the prevalence of metabolic syndrome. We used author reported definitions (according ATP III &IDF) (Table 1). Secondary outcome was factors associated with metabolic syndrome among type 2 diabetes mellitus patients. We were used unpublished articles to identify any potential studies that might have been missed from our search.

**Table 1:** Definition of Metabolic syndrome by using NCEP/ATPIII and IDF criteria

	NCEP/ATPIII	IDF
Absolutely required	None	Central obesity (waist circumference): 94 cm (M) and ≥ 80 cm (F)
Criteria	Any three of the five criteria below	Obesity, plus two of the four criteria below
Obesity	Waist circumference: >40 inches (M) and > 35 inches (F)	Central obesity already required
Hyperglycemia	Fasting glucose > 100 mg/dl or Rx	Fasting glucose > 100 mg/dl
Dyslipidemia	TG > 150 mg/dl or Rx	TG > 150 mg/dl or Rx
Dyslipidemia (second, separate criteria)	HDL-C: <40 mg/dl (M) and < 50 mg/dl (F) or Rx	HDL-C: <40 mg/dl (M) and < 50 mg/dl (F) or Rx
Hypertension	> 130 mmHg systolic or > 85 mmHg diastolic or Rx	> 130 mmHg systolic or > 85 mmHg diastolic or Rx

^*^Rx, pharmacologic treatment; *NCEP/ATPIII, National Cholesterol Education Program–Adult Treatment Panel III; *IDF, International Diabetes Federation; *TG, triglyceride; *HDL-C, high-density lipoprotein cholesterol

#### Exclusion criteria

We excluded reviews, case reports, case series, qualitative studies, and opinion articles. We were exclude abstract-only papers.

### Data extraction and quality assessment

Data extraction was done independently by the two reviewers in a pre-piloted data extraction form created in MS Excel. Any discrepancies in the extracted data were resolved by consensus or discussion with a third reviewer. The following details will be extracted from each study:- details of the study (first author’s last name, year of publication), study region, study design, sample size, Prevalence of metabolic syndrome, associated factors.

The Joanna Brigg Institute’s quality evaluation criteria’s (JBI) were used to evaluate the studies’ quality. We assessed each of the chosen publications using the JBI assessment checklist. Research with a quality score of at least 50% was deemed to be of high quality.

### Data analysis

Version 17 of STATA was used to analyse the retrieved data after they were imported into Microsoft Excel. To get a general summary estimate of the prevalence across trials, a random-effects model was employed. We employed point estimate with a 95% confidence interval. Sensitivity analysis was used to evaluate each study’s contribution to the outcome by eliminating each one individually. Using Egger’s test, the existence of publication bias was evaluated. The Cochran’s Q statistic and I2 statistics were used to assess the heterogeneity of the studies. Moreover, meta-regression has been conducted that represents linear predictions for the metabolic syndrome among type 2 diabetes mellitus patients prevalence as a function of published year. Subgroup analysis was performed based on study region and study subjects since there was unexplained significant heterogeneity.

### Publication bias

**Fig. 2:**
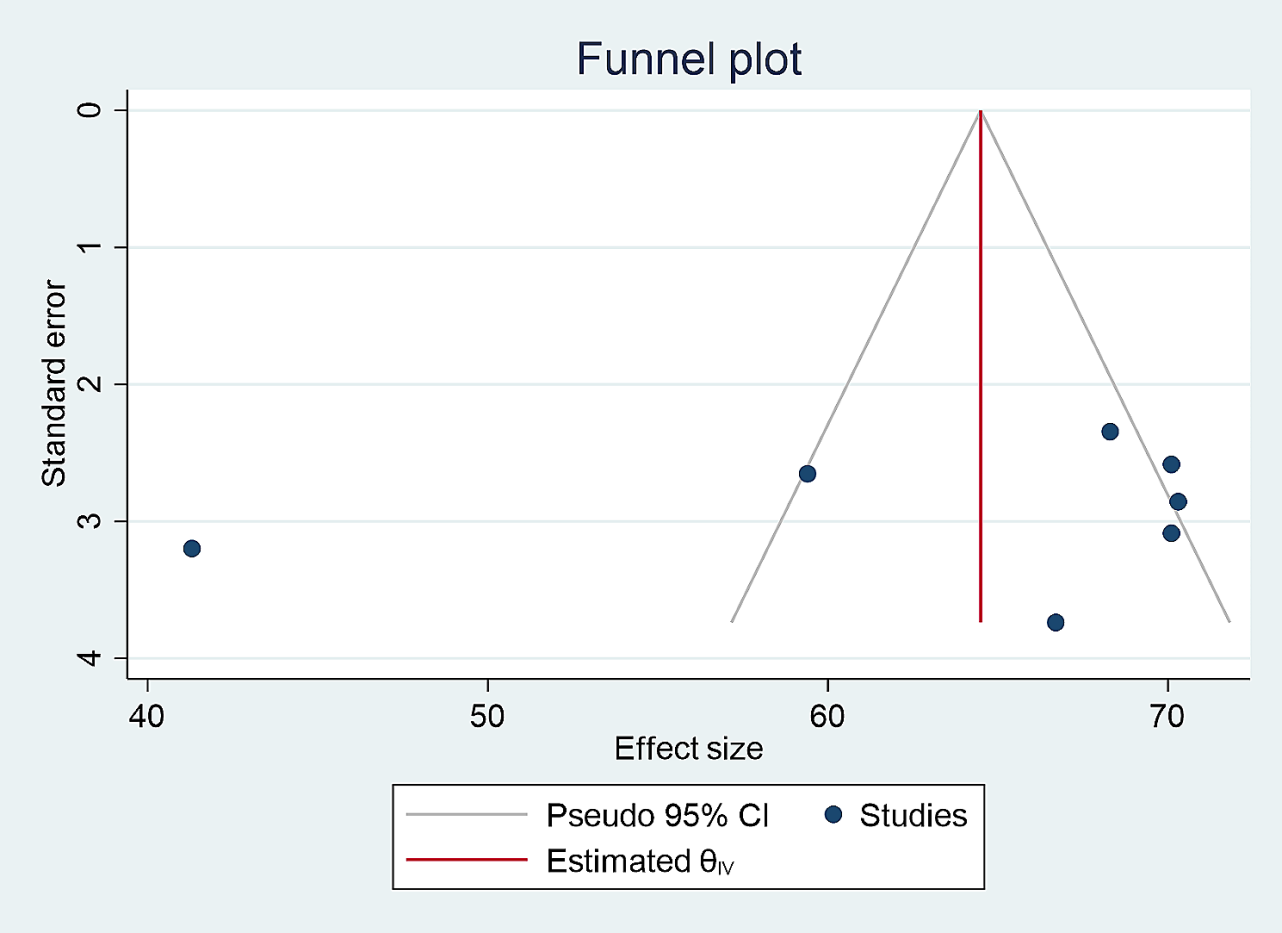
Forest plot showing the pooled prevalence of metabolic syndrome among type II Diabetes mellitus patients in Ethiopia (according to NCEP ATP III Criteria)

**Fig. 3:**
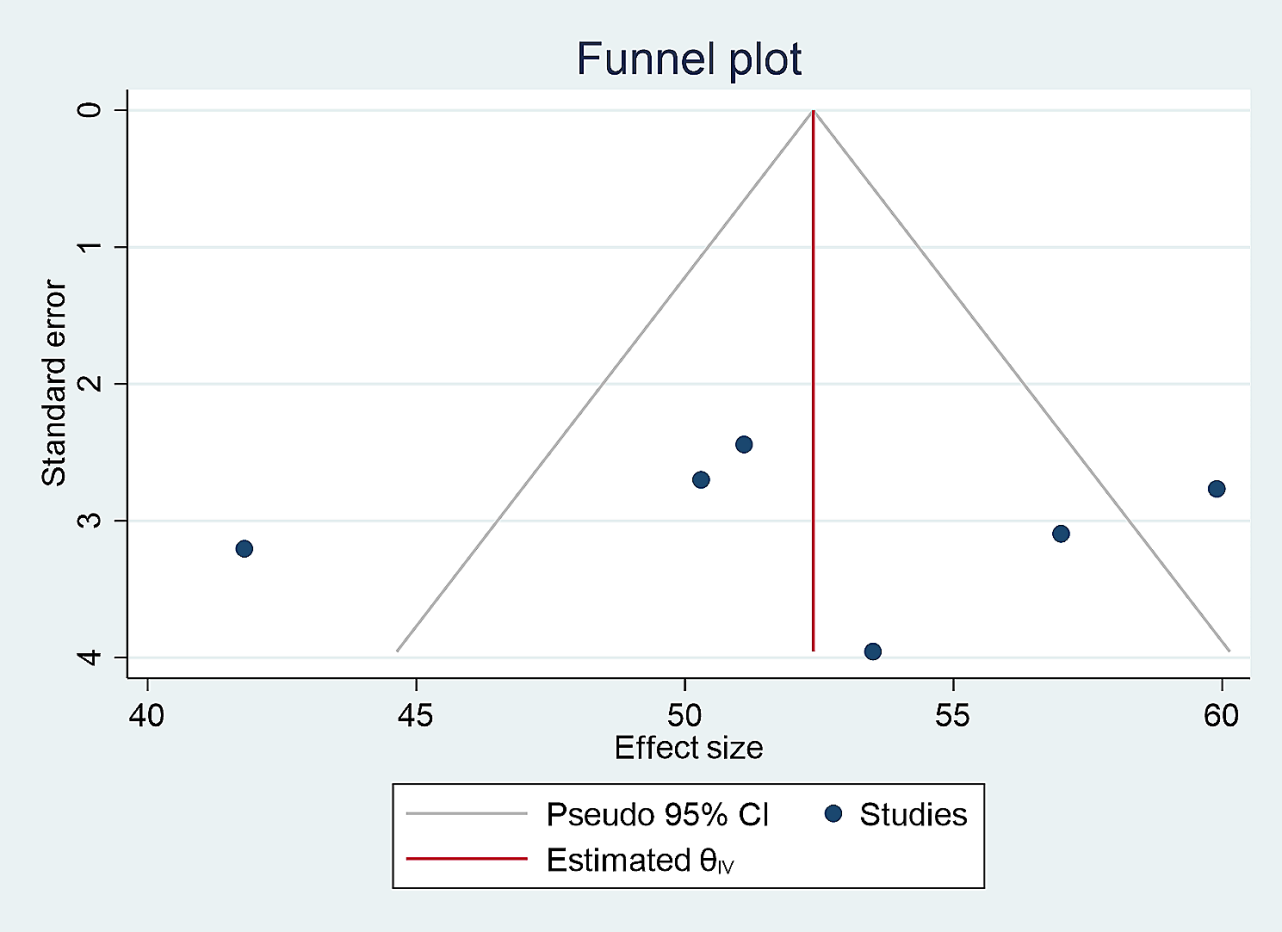
Forest plot showing the pooled prevalence of metabolic syndrome among type II Diabetes mellitus patients in Ethiopia (according to IDF Criteria)

Funnel plot and Egger’s test was used to assess publication bias and a P-value of less than 0.05 was used to declare the publication bias. The included studies were assessed for potential publication bias and separate analyses were done based on IDF and NCEP/ATPIII criteria (p values were 0.58 and 0.88, respectively) which indicated the absence of publication bias (Figs. 2 and 3).

## Result

### Characteristics of included studies

The title and abstract screening of 300 potential articles yielded 102 that were included relevant to the topic of interest; the full-text screening of 50 of these articles indicated their eligibility for full-text assessment; 8 of these articles, involving 2375 study participants, were found to be eligible for systematic review and meta-analysis. Based on the NCEP/ATPIII and IDF criteria, the prevalence of metabolic syndrome in patients with type 2 diabetes mellitus was assessed among the Ethiopian population of different study participants. Five studies reported the prevalence of metabolic syndrome among type 2 diabetes mellitus based on both IDF and NCEP/ATPIII criteria, whereas seven studies based on NCEP/ATPIII criteria only and six studies by IDF criteria only (Table 2).

**Table 2:** Baseline characteristics and outcome of included studies

ID	First Author Name/Publication year	Study Region	Study design	Sample size	Studied population	Proportion NCEP-ATP III(%)	Proportion % IDF
1	Zerga AA /2020	Amhara	cross-sectional	343	343	59.4	50.3
2	Belete B et al. /2018	Amhara	cross-sectional	159	159	66.7	53.5
3	Gemeda D et al/2022	Oromia	cross-sectional	422	394	68.3	
4	GebremeskelGG et.al/2019	Tigray	cross-sectional	419	419		51.1
5	Birarra MK/2018	Amhara	cross-sectional	256	256	70.3	57
6	Woyesa et al./2017	SNNRP	cross-sectional	220	220	70.1	
7	W Bizuaye et al/2022	SNNRP	cross-sectional	319	314	70.1	59.9
8	Charkos TG/2023	Oromia	cross-sectional	237	237	41.3	41.8

### Prevalence of metabolic syndrome among type 2 diabetes mellitus patients using IDF and NCEP ATP III Criteria

**Fig. 4:**
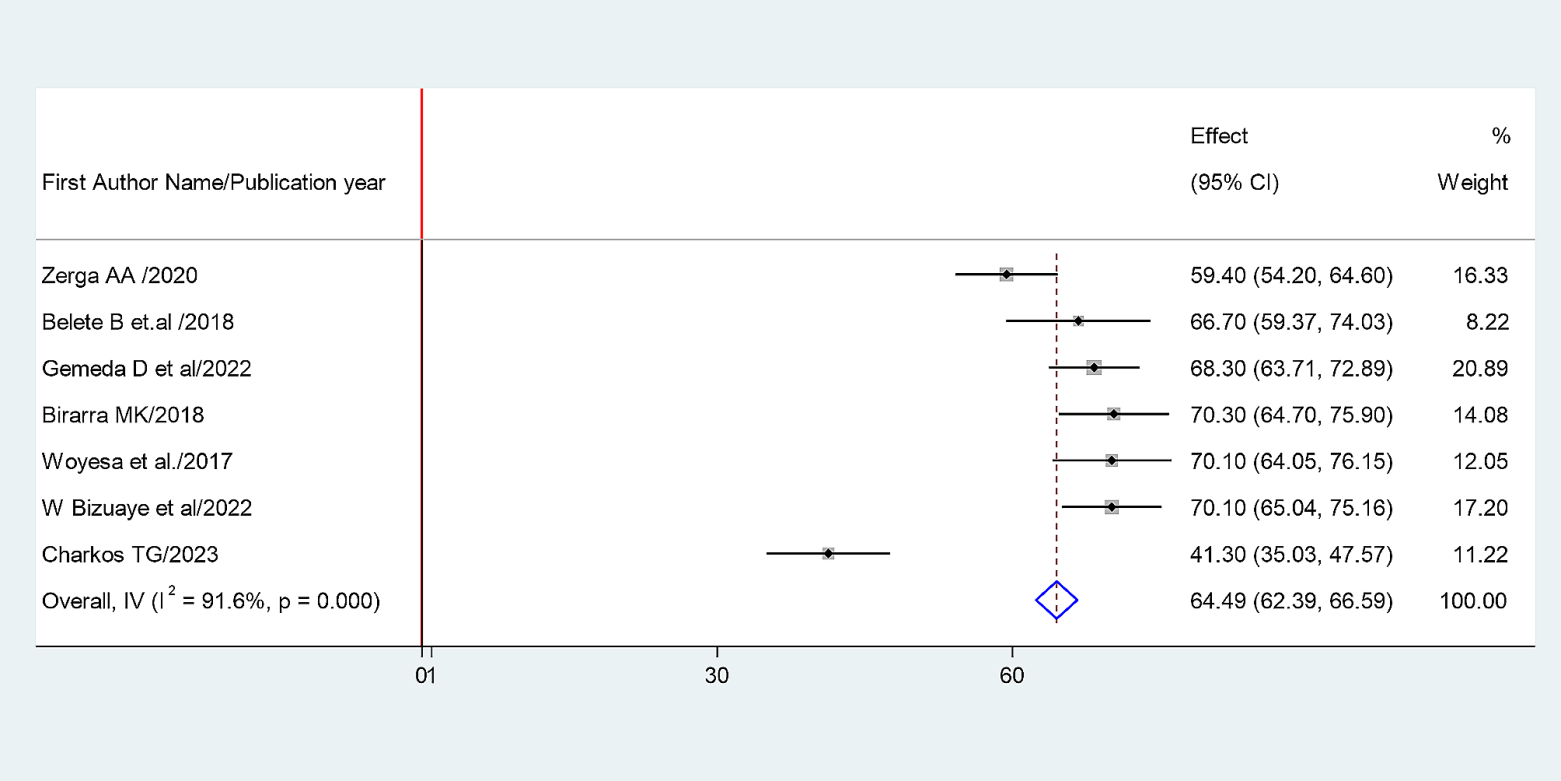
Sub group analysis based on study region using NCEP ATP III Criteria

**Fig. 5:**
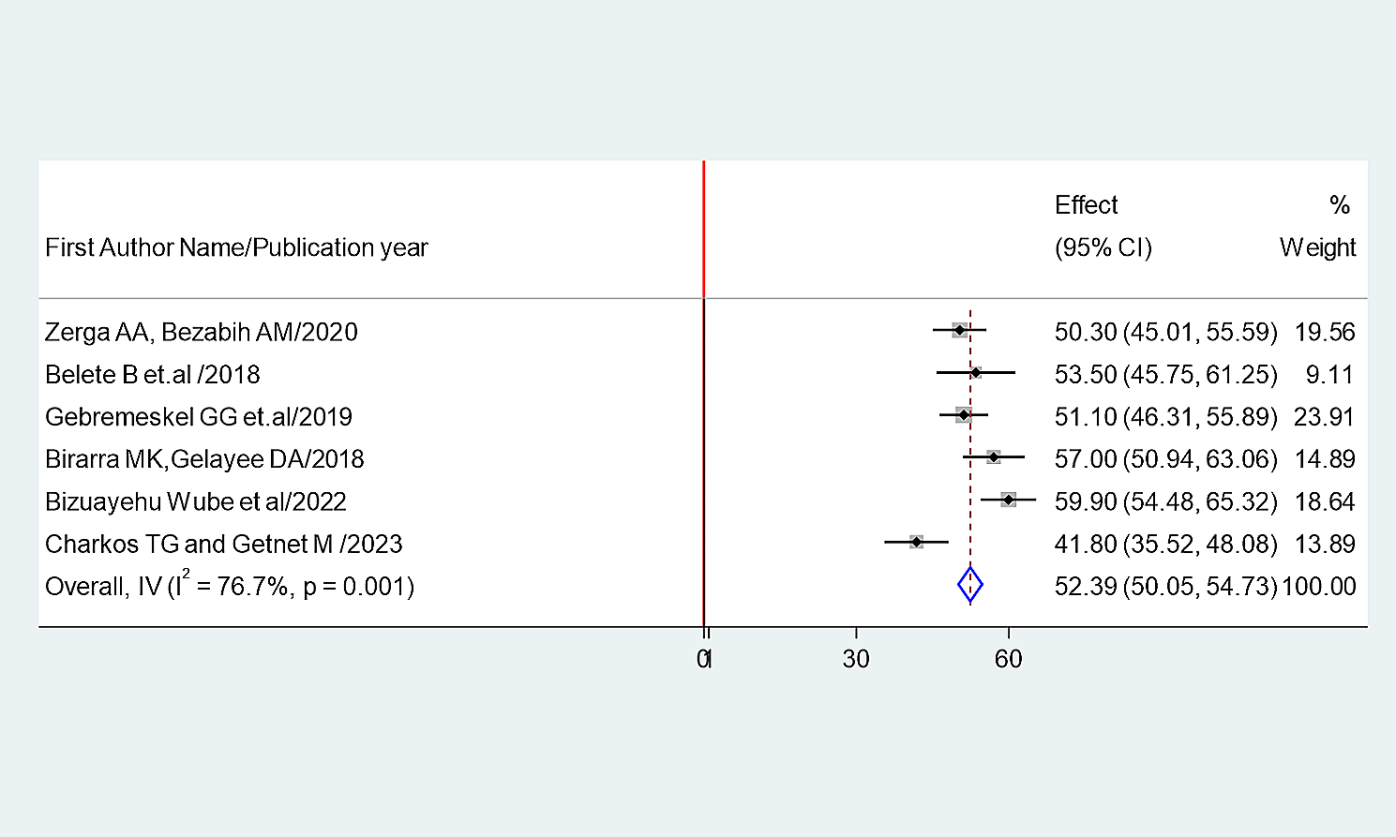
the pooled odds ratio of the association between sex and prevalence of metabolic syndrome among type II diabetes mellitus patients

**Fig. 6:**
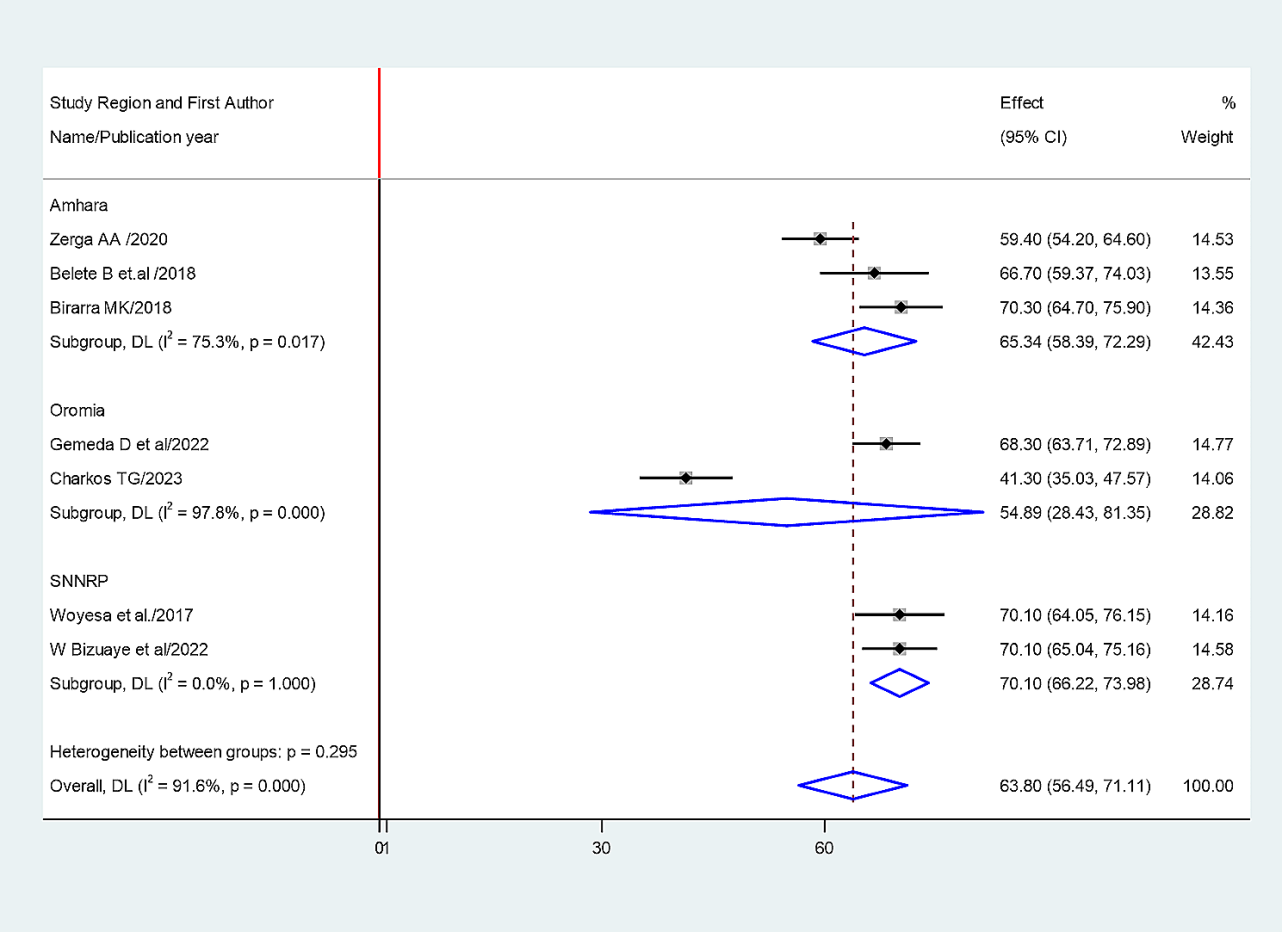
The pooled odds ratio of the association between BMI and prevalence of metabolic

The random-effects model was applied since the heterogeneity index of the studies were significant. The pooled prevalence of metabolic syndrome was found to be 64.49% (95% CI: 62.39, 66.59) by using NCEP/ATP III (Figs. 4) and 52.38% (95% CI: 50.05, 54.73) by using IDF criteria (Fig. 5). Subgroup analysis based on the study region using NCEP/ ATP III showed that the weighted pooled prevalence was 63.79% (95% CI: 56.48, 71.11) among type 2 diabetes patients (Fig. 6). Using IDF criteria, subgroup analysis based on the study region showed that the weighted pooled prevalence was 52.23% (95%CI: 47.37, 57.22).

### Factors associated with metabolic syndrome among type II DM

#### Association between sex and prevalence of metabolic syndrome

**Fig. 7:**
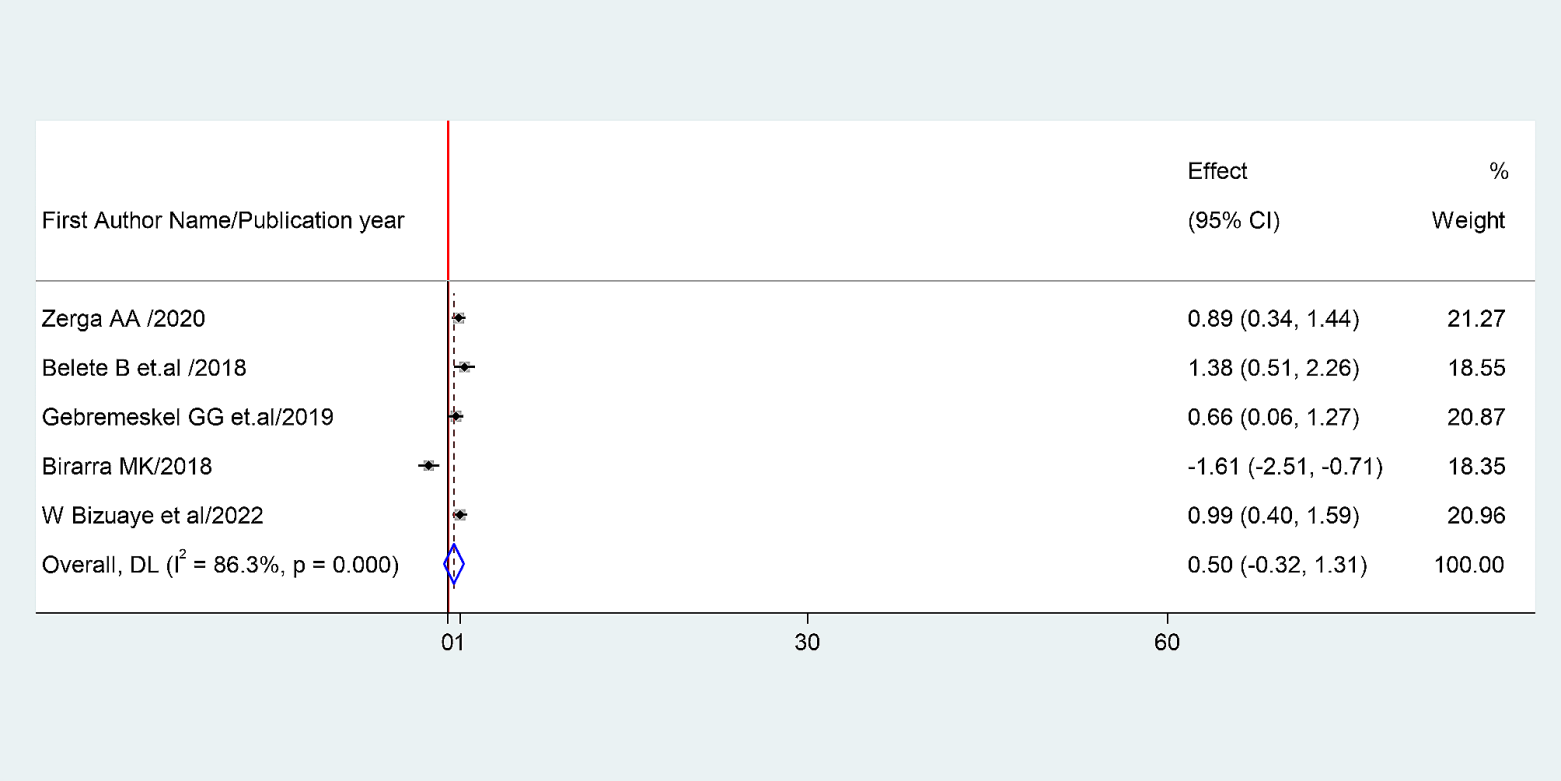
Funnel plot for prevalence of metabolic syndrome according to NCE ATPIII

The association between being female and prevalence of metabolic syndrome was examined based on the finding from five studies (1, 2, 4, 5 and 7). The pooled odds ratio (AOR: 0.5, 95% CI: -0.32-1.31) showed that prevalence of metabolic syndrome associated with being female. The studies showed very high heterogeneity (I²=86.3% and *P* = 0.00) (Fig. 7). Hence, a random effects model was employed to do the final analysis.

#### Association between BMI and prevalence of metabolic syndrome

**Fig. 8:**
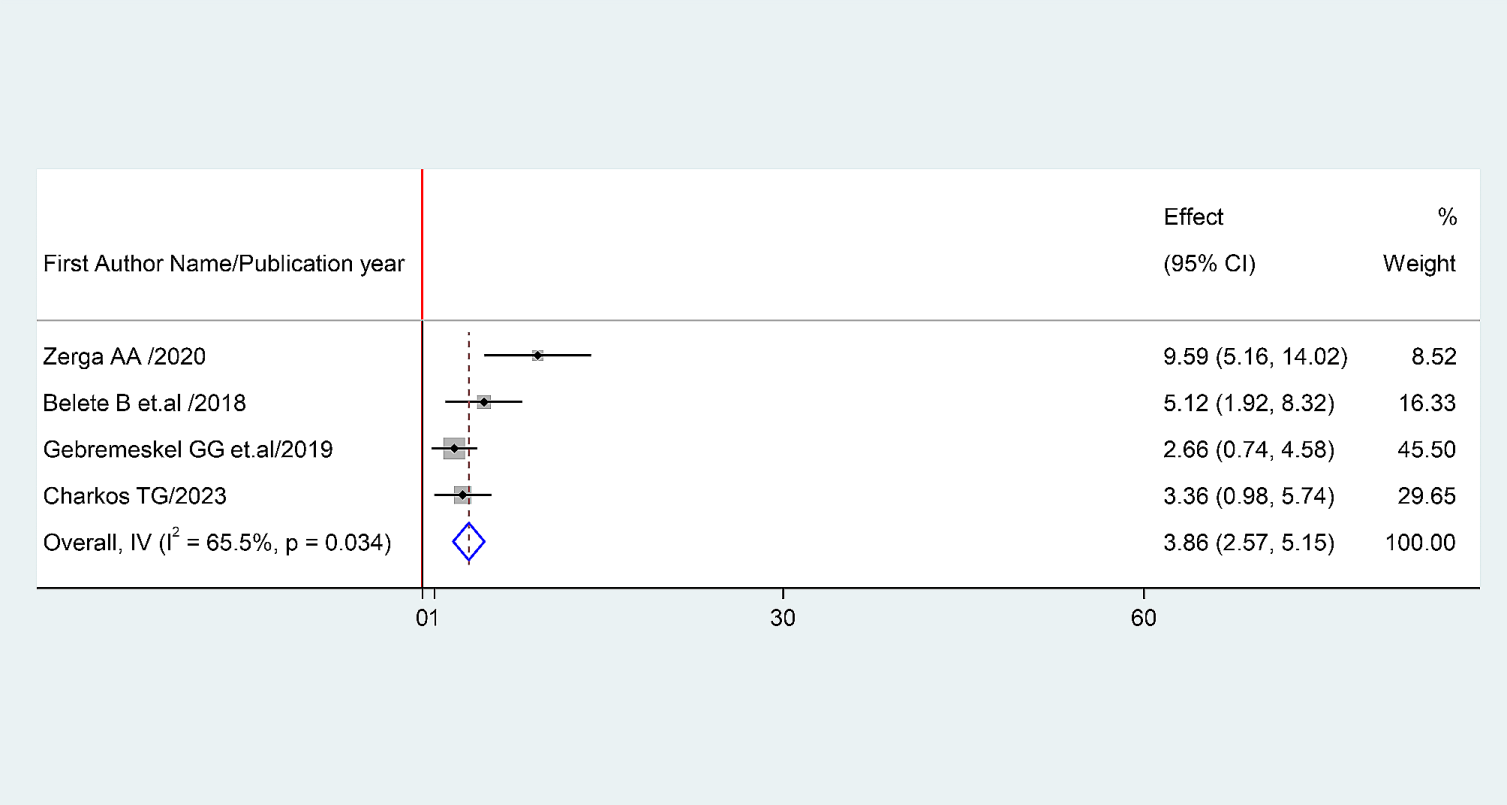
Funnel plot for prevalence of metabolic syndrome according to IDF

The association between BMI and prevalence of metabolic syndrome was examined based on the finding from four studies (1, 2, 4 and 8). The pooled odds ratio (AOR: 3.86, 95% CI: 2.57–5.15) showed that prevalence of metabolic syndrome associated with BMI. The studies showed high heterogeneity (I²= 68.2% and *P* = 0.034) (Fig. 8). Hence, a random effects model was employed to do the final analysis.

### Sensitivity analysis

**Fig. 9:**
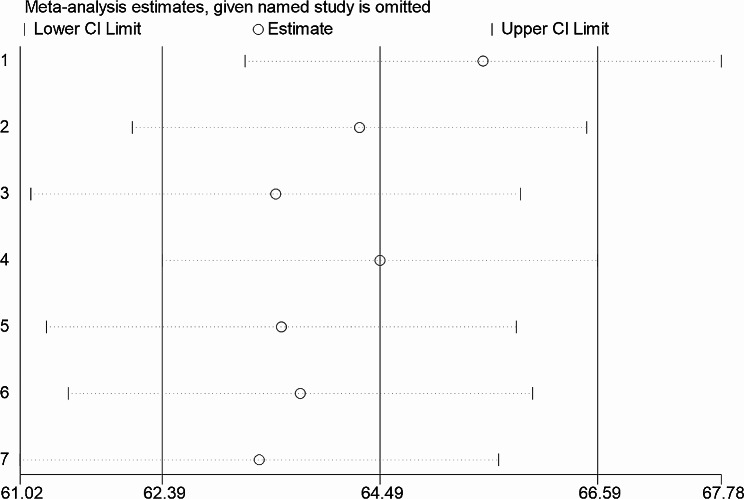
Sensitivity analysis based on NCEP/ATP III diagnostic criteria

**Fig. 10:**
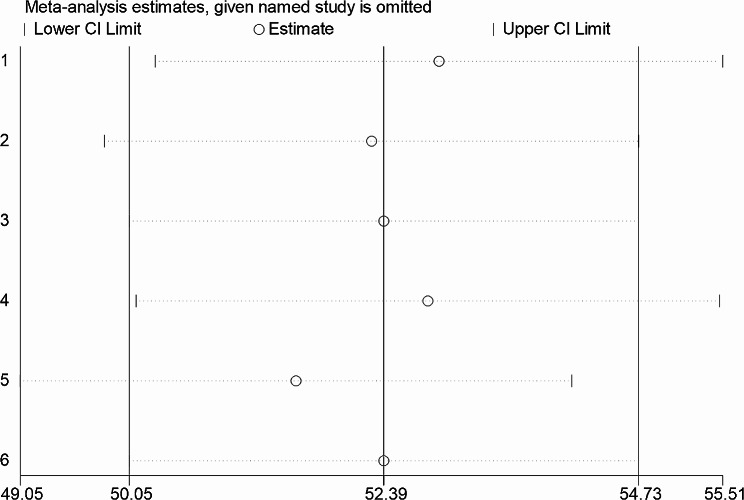
Sensitivity analysis based on IDF diagnostic criteria

Sensitivity analysis was carried out by gradually removing each research from the analytic process according to the two provided diagnostic criteria (NCEP/ATP III and IDF) in order to evaluate the impact of each study on the pooled estimated prevalence of metabolic syndrome among type II diabetes mellitus patients. The result showed that excluded studies led to significant changes in the shared estimation of the prevalence of metabolic syndrome (Figs. 9 and 10).

## Discussion

This systematic review and meta- analysis study provides evidence of an estimated pooled prevalence of metabolic syndrome among type II diabetes mellitus patients of the Ethiopian population. According to this review, the combined pooled prevalence of Metabolic syndrome among type II diabetes mellitus patients were 64.49% (95% CI: 62.39, 66.59) and 52.38% (95% CI: 50.05, 54.73) by using NCEP/ATP III and IDF criteria, respectively.

The finding of this study similar with the study conducted in African populations, which reported that the prevalence of metabolic syndrome among type 2 diabetes mellitus was 66.9%(95%CI: 60.3–73.1) [9]. In addition, the study which was done in sub-Saharan African countries in line with this finding which reports that prevalence of metabolic syndrome among type II diabetes melituse patients were 64.8% (95% CI: 54.74, 74.86) and 57.15% by using NCEP/ATP III and IDF criteria, respectively.

Furture more, the study which was done in Sub-Saharan Africa reported that, among others sub-Saharan Africa countries the prevalence of metabolic syndrome was highest in Ethiopia, (61.14%, 95% CI: 51.74, 70.53), which is almost similar with our study findings [8].

Similarly the study conducted in Ethiopian population showed that the weighted pooled prevalence of metabolic syndrome among type II diabetes mellitus patient was 63.78% (95% CI: 56.17, 71.40) [7].

This study result supported by the fact that metabolic syndrome has been associated with type 2 diabetes due to its high prevalence worldwide since it is both related to the increase in obesity and a sedentary lifestyle. Several studies suggest that individuals with Metabolic syndrome are 5 times more likely to develop type 2 diabetes [10].

This meta-analysis assessed factors determining metabolic syndrome among type II diabetes mellitus patients. The current meta-analysis demonstrated that the prevalence rate of metabolic syndrome was higher in females compared to that in males. This has been shown in all the Middle Eastern countries, and the prevalence was much higher among women than men [6]. The prevalence rate of metabolic syndrome associated with the individual’s body mass index.

This study has implications for clinical practice. Determining the prevalence of metabolic syndrome among type 2 diabetic patients is critical to guide healthcare professionals to minimize the risk of metabolic syndrome by providing guidance to the patient who has undergone diabetic care follow up. Moreover, it gives information about the burden and public health impact of metabolic syndrome for possible consideration during routine diabetic patient care.

This meta-analysis study has its own limitations that should be considered in the future research. Few studies are included due to limited research in Ethiopia which makes it difficult to generalize the findings to all type 2 diabetic patients in the countery and which makes the discussion part more shallow.

## Conclusion

In conclusion, according to this systematic review the prevalence of metabolic syndrome among type II patient is high in Ethiopia and recommends an urgent attention from both the clinical and public health viewpoint Therefore, policymakers, clinicians, and concerned stakeholders shall urge effective strategies in the control, prevention, and management of metabolic syndrome among type II diabetes mellitus. In addition country context-specific preventive strategies should be developed to reduce the burden of metabolic syndrome.

## Data Availability

The data used to support the findings of this study are available from the corresponding author upon request.

## Abbreviations

CVD: Cardiovascular Disease
NCEP/ATP III: National Cholesterol Education Program–Adult Treatment Panel III
IDF: International diabetes federation
PRISMA: Preferred Reporting Items for Systematic Reviews and Meta-Analyses
WHO: World Health Organization
MS: Metabolic syndrome

## References

[1] Saklayen MG (2018). The global epidemic of the metabolic syndrome. Curr Hypertens Rep.

[2] Grundy et al. Definition of metabolic syndrome, January 27, 2004, 10.1161/01.CIR.0000111245.75752.C6.

[3] Shin JA, Lee JH, Lim SY, Ha HS, Kwon HS, Park YM, Lee WC, Kang MI, Yim HW, Yoon KH, Son HY (2013). Metabolic syndrome as a predictor of type 2 diabetes, and its clinical interpretations and usefulness. J Diabetes Investig.

[4] Abagre (2022). Determinants of metabolic syndrome among patients attending diabetes clinics in two sub-urban hospitals: Bono Region, Ghana. BMC Cardiovasc Disord.

[5] Lira Neto JCG, Xavier MA, Borges JWP, Araújo MFM, Damasceno MMC, Freitas RWJF (2017). Prevalence of metabolic syndrome in individuals with type 2 diabetes Mellitus. Rev Bras Enferm [Internet].

[6] Samrawit Solomon and Wudeneh Mulugeta (2019). Disease burden and associated risk factors for metabolic syndrome among adults in Ethiopia. BMC Cardiovasc Disord.

[7] Sintayehu Ambachew A, Endalamaw A, Worede Y, Tegegne M, Melku B, Biadgo. The Prevalence of Metabolic Syndrome in Ethiopian Population: A Systematic Review and Meta-analysis, *Journal of Obesity*, vol. 2020, Article ID 2701309, 14 pages, 2020. 10.1155/2020/2701309. 10.1155/2020/2701309 PMC7803160 33489358

[8] Shiferaw WS, Akalu TY, Gedefaw M, Anthony D, Kassie AM. Worku Misganaw Kebede, Henok Mulugeta, Getenet Dessie, Yared Asmare Aynalem, Metabolic syndrome among type 2 diabetic patients in Sub-Saharan African countries: A systematic review and meta-analysis,Diabetes & Metabolic Syndrome: Clinical Research & Reviews, Volume 14, Issue 5, 2020, Pages 1403–1411, ISSN 1871–4021, 10.1016/j.dsx.2020.07.013. 10.1016/j.dsx.2020.07.013 32755843

[9] Bowo-Ngandji A (2023). Prevalence of the metabolic syndrome in African populations: a systematic review and meta-analysis. PLoS ONE.

[10] Regufe VMG, Pinto CMCB, Perez PMVHC (2020). Metabolic syndrome in type 2 diabetic patients: a review of current evidence. Porto Biomed J.

[11] Page MJ, McKenzie JE, Bossuyt PM, Boutron I, Hoffmann TC, Mulrow CD (2021). The PRISMA 2020 statement: an updated guideline for reporting systematic reviews. BMJ.

